# Isolation of bergenin from the root bark of *Securinega virosa* and evaluation of its potential sleep promoting effect

**Published:** 2015

**Authors:** Mohammed Garba Magaji, Aliyu Muhammad Musa, Musa Ismail Abdullahi, Jamilu Ya’u, Isa Marte Hussaini

**Affiliations:** 1 *Department of Pharmacology and Therapeutics, Ahmadu Bello University, Zaria-Nigeria*; 2 *Department of Pharmaceutical and Medicinal Chemistry, Ahmadu Bello University, Zaria-Nigeria*; 3 *Department of Pharmaceutical and Medicinal Chemistry, Usmanu Danfodiyo University, Sokoto-Nigeria*; 4 *Department of Pharmacology, University of Maiduguri, Maiduguri-Nigeria*; 5 *Department of Pathology, University of Virginia, USA*

**Keywords:** *Securinega virosa*, *Bergenin*, *Sleep*, *Isocoumarin*

## Abstract

**Objectives:**

*Securinega virosa *Roxb (Ex Willd) Baill (Euphorbaiceae) root bark has been reportedly used in African traditional medicine in the management of mental illnesses. Previously, the sleep-inducing potential of the crude methanol root bark of *Securinega virosa* extract and its butanol fraction have been reported. The study aimed to isolate and characterize the bioactive constituent that may be responsible for the sleep inducing property of the root of the plant.

**Materials and Methods:**

The phytochemical investigation of the *S. virosa* root bark was carried out leading to the isolation of a compound from the butanol-soluble fraction of the methanol extract. The structure of the compound was elucidated on the basis of its spectral data, including IR, 1D and 2D NMR, mass spectrometry as well as X-ray diffraction analysis*.* The compound was investigated for sleep-inducing potential using diazepam-induced sleeping time test and beam walking assay in mice.

**Results:**

This is the first report on the isolation of bergenin from the root of the plant. It significantly decreased the mean onset of sleep [F (2, 15) =7.167; p< 0.01] at the dose of 10 mg/kg, without significantly affecting the total sleep duration [F (2, 15) = 0.090, p=0.914]. Conversely, it did not significantly affect the number of foot slips at the doses of 5 and 10 mg/kg tested.

**Conclusion:**

Bergenin isolated from the root bark of *S. virosa* possesses sleep-inducing property and could be partly responsible for the sedative potential of the root of *S. virosa*.

## Introduction

According to the World Health report (WHO, 2001 ▶), approximately 450 million people suffer from a mental or behavioral disorders, yet only a small minority of them receive even the most basic treatment. This amounts to 12.3% of the global burden of disease, and is speculated to rise to 15% by 2020 (Reynolds, 2003 ▶). Insomnia is a common complaint of inadequate sleep affecting 15-40% of the world population, which complicates several disorders and of which less than 15% receives appropriate treatment (Jiang et al., 2007 ▶). The statistics are thought to be higher due to existence of compelling evidences of under-diagnosis, under-recognition and under-treatment of the condition (Najib, 2006 ▶). Insomnia is both a risk factor and precursor of behavioral disorder such as depression and, therefore, its effective treatment is essential in the prevention of the major depressive illnesses (Forde and Kamerow, 1989 ▶; Eaton et al., 1995 ▶). The existing agents used in the management of insomnia which include the benzodiazepines and non-benzodiazepine drugs are associated with untoward effects such as day time fatigue, cognitive impairment and physical dependence. This has necessitated the affected patients to patronize herbal remedies with claims of lower incidence of adverse effects. 

 The reliance of patients on herbal remedies for the management of their neuropsychiatric disorders is one of the several reasons encouraging ethnopharmacological researches towards the development of potential therapeutic agents with better safety and efficacy profiles for the management of neurological conditions (Zhang, 2004 ▶). The potentials of a number of these medicinal plants in the management of neuropsychiatric disorders have been investigated using batteries of behavioral models. Some of these medicinal plants are promising sources of therapeutic agents in the management of CNS disorders.

S*ecurinega virosa *is one of the African medicinal plants described as a true “cure all”, of which all parts are used as remedies, particularly the root. The common vernacular names in Nigeria include “tsuwaawun karee, gussu, gwiiwar karee” (Hausa), “iranje” (Yoruba), “njisinta” (Ibo), “shim shim” (Kanuri), “kartfi-kartfi” (Shuwa arabs) and “camal, cambe, came” (Fulani) (Neuwinger, 1996 ▶). The decoction of the root with other plant is used in northern Nigeria for the treatment of mental illness. The crude methanolic root bark extract of the plant has been reported to possess sedative activity in laboratory animals (Magaji et al., 2008 ▶). Similarly, the neuropharmacological activities of various fractions of the methanol root bark extract of the plant have been reported (Magaji et al., 2013 ▶; Magaji et al., 2014a, b ▶). A number of alkaloids have been isolated from the root bark of *S. virosa*. These include hordenine, norsecurinine (Iketubosin and Mathieson, 1963 ▶), dihydronorsecurinine, viroallosecurinine (Saito et al., 1964 ▶) and 14, 15-epoxynorsecurinine (Dehmlow et al., 1999 ▶). Friedelin, epifriedelanol, stigmasterol and betulinic acid were isolated from the leaves and twig of the plant (Monkodkaew et al., 2009 ▶). The aim of the present study, therefore, is to isolate the potential sleep-inducing compound from the methanolic extract of root bark of *S. virosa.* In this study, we report, for the first time, the isolation of bergenin from the root of *S. virosa* and its sleep-inducing potential. 

## Materials and Method

**Collection and identification of plant material**

The whole plant, *S. virosa* Roxb (ex Willd) Baill (Euphorbiaceae) was collected from Basawa town, in Sabon-Gari Local Government Area of Kaduna State, Nigeria, in November 2008. The plant was identified and authenticated by Messr Umar Galla of the Herbarium Section in the Department of Biological Sciences, Ahmadu Bello University, Zaria-Nigeria, by comparing with existing specimen (NO 918). The root was cleaned and the bark removed. The root bark was air dried under shade until a constant weight was obtained. It was then crushed into coarse powder with a pestle and mortar.

**Animals**

Male Swiss Albino mice weighing 18-22 g (7-8 weeks old), were obtained from the Animal House Facility of the Department of Pharmacology and Therapeutics, Ahmadu Bello University Zaria, Nigeria. The animals were maintained in a well-ventilated room in the animal house. They were fed on standard laboratory animal feed and water *ad libitum.* All experiments performed on laboratory animals in this study were in accordance with Ahmadu Bello University Research policy as well as ethic and regulations governing the care and use of experimental animals as contained in “Principles of laboratory animal care” published by the National Institute of Health (NIH Publication No. 85-23, revised, 1996). The experiments were conducted in quiet laboratory between hours of 9:00 and 16:00.

**Extraction and fractionation**

The powered root bark (1000 g) was extracted with 2 l methanol (absolute) for 72 hours using soxhlet extraction apparatus. The solvent was evaporated on a water bath at 40ºC to give a dark brownish residue (Average yield: 9.82 %w/w) which was subsequently referred to as crude methanolic root bark extract. Then, 50 g of the crude methanolic root bark extract was solubilized in distilled water and filtered. The filtrate was successively partitioned with petroleum ether, chloroform, ethyl acetate and n-butanol to yield petroleum ether, chloroform, ethyl acetate and n-butanol-soluble fractions, respectively. 

**Chromatrographic techniques**

Three grams (3 g) of butanol fraction was chromatographed on silica gel column eluting with ethylacetate (100%), ethyl acetate/ methanol mixtures (95:5, 90:10, 80:20, 70:30 and 50:50) and methanol 100% as solvent systems to give three major fractions when pooled together based on similarity in their thin layer chromatographic (TLC) profile coded Pooled fraction A (PFA), Pooled fraction B (PFB) and Pooled fraction C (PFC). Repeated Sephadex LH-20 gel filtration chromatography using methanol as eluent led to the isolation of a compound coded MAG from the most active fraction, PFA. Thin layer chromatography (TLC) was performed using silica gel 60 F_254_ (Merck); Column chromatography was performed using Merck silica gel (60–120) mesh and gel filtration chromatography was performed using Sephadex LH-20 (Sigma, USA). Spots on TLC plates were visualized by spraying 10% H_2_SO_4_ followed by heating at 100 °C for 5min or with Gibbs reagent. 

**Spectral analysis**

NMR-spectra were recorded on a Bruker AVANCE spectrometer (400 MHz) for ^1^H and (100 MHz) for ^13^C-NMR using residual solvent peak as internal standard; deuterated methanol was used as solvent and chemical shift values () were reported in parts per million (ppm). The IR spectrum was measured on a Shimadzu FT-IR8 400S Fourier Transform Infrared spectrophotometer. The UV spectrum was recorded by a Hitachi U-3200 spectrophotometer. Melting point was determined using a Yanaco MP-400 micro melting point apparatus. X-ray crystallography was obtained at low temperature from ethyl acetate crystals. The results obtained were compared with those reported in the literature and databases.

**Pharmacological studies**

*Diazepam-induced sleep in mice*

The method described by Beretz et al. (1978) ▶ and modified by Rakotonirina et al. (2001) ▶ was adopted. Initially, the butanol soluble fraction (75, 150 and 300mg), PFA (2.5, 5 and 10 mg/kg), PFB (2.5, 5 and 10 mg/kg) and PFC (2.5, 5 and 10 mg/kg) were subjected to the diazepam induced sleep test. For the isolated compound, 

mice of either sex were randomly divided into three groups each containing 6 mice. The first group received normal saline (10 ml/kg). The second and third groups received bergenin 5 and 10 mg/kg, respectively. Thirty minutes post-treatment, diazepam at a dose of 25 mg/kg body weight was administered to the mice. The mice were placed individually in cages. The onset and the duration of sleep were determined for each animal. Loss of righting reflex was considered as the criterion for sleep (Rolland et al., 1991 ▶) while the interval between the loss and the recovery of straightening was regarded as the duration of sleep (Fujimori, 1965 ▶). 

**Mouse Beam walking Assay in mice**

The method previously described by Stanley et al. (2005) ▶ was adopted with slight modification (Magaji et al., 2008 ▶). Mice were trained to walk from a start platform along a ruler (80 cm long, 3 cm wide) elevated 30 cm above the bench by metal support to a goal box. Three trials were performed for each mouse. Trials were designed in a way that the mouse tested would be aware of the presence of a goal box that could be reached. The goal box was a Perspex glass cage (with wood chippings beddings) with a small hole at the bottom. The mice that successfully walked along the ruler were randomly grouped into five groups each containing six mice. The first group received normal saline (10 ml/kg), i.p. The second and third groups received bergenin 5 and 10 mg/kg, respectively. The fourth group received diazepam (2 mg/kg body weight). The beam was made of wood, 8 mm in diameter, 60 cm long and elevated 30 cm above the bench by metal support. Thirty (30) minutes post- treatment, each mouse was placed on the beam at one end and allowed to walk to the goal box. Mice that fell were returned to the position they fell from, with a maximum time of 60 seconds allowed on beam. The number of foot slips (one or both hind limb slipping from the beam) was recorded with the aid of a tally counter. The time taken to complete the task was also recorded. 

**Statistical analysis**

Results of the pharmacological studies were expressed as mean ± standard error of mean. Statistical analysis was performed by One-Way analysis of variance (ANOVA); when a statistically significant result was obtained with ANOVA, a post-hoc Dunnets t-test was performed for multiple comparisons. Values of P<0.05 were considered significant.

## Results

**Figure 1:**
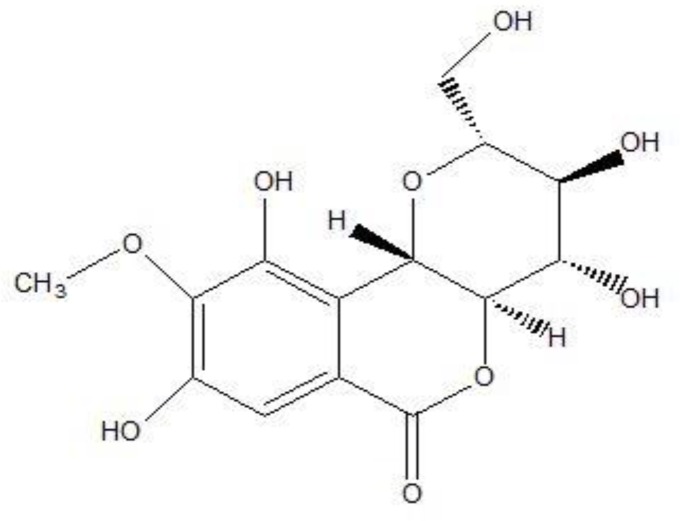
Bergenin

MAG was obtained as colourless crystals (21.5mg) and was characterized by comparison with spectral data reported in the literature; m.p 192–195°C; UV (MeOH) λ_max_: 265, 320nm; IR (KBr): *νmax *3350, 1680 cm^−1^ representing OH and carbonyl functional groups respectively (Dung et al., 2003). HREIMS measurements yielded an ion [M-H]^− ^(*m/z*=327) corresponding to C_14_H_16_O_9 _and the fragmentation pattern is in agreement with that of bergenin (Dung et al., 2003 ▶; Li et al., 2013 ▶). The 1 & 2D NMR experiments including ^1^H,^ 13^C,^ 1^H-^1^H,

HSQC, HMBC and NOIESY data were in agreement with those reported in the literature (Dung et al., 2003 ▶; Nasser et al., 2009 ▶; da Silva et al., 2009 ▶; Nunomura et al., 2009 ▶) for bergenin (Figure 1). 

**Figure 2:**
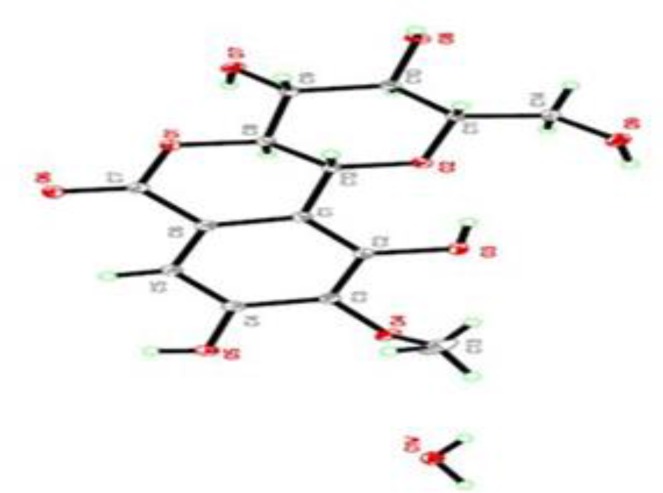
View of bergenin showing the x-ray analysis of crystal monohydrate

The x-ray crystallographic analysis of MAG established the stereochemistry (Figure 2) as previously reported (Caldas et al., 2002 ▶). 

**Pharmacological Studies**

**Figure 3:**
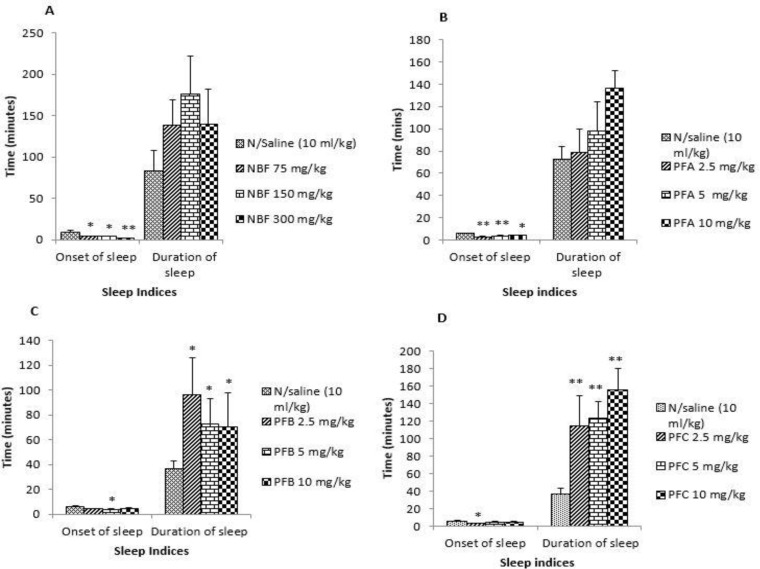
Effect of n-butanol soluble fraction of methanolic extract of root bark of *Securinega virosa *and its various fractions on diazepam-induced sleep in mice; Data presented as mean ± SEM; NBF (n-butanol fraction); PFA (Pooled fraction A) PFB (Pooled fraction B); PFC (Pooled fraction C); *p < 0.05 and **p < 0.001 compared to control; n ═ 6.

**Figure 4:**
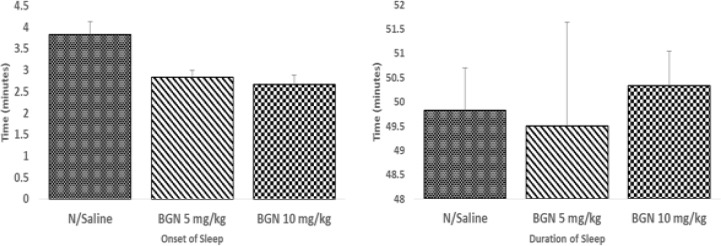
Effect of bergenin (BGN) on diazepam-induced sleep in mice *p< 0.05 compared to control; n ═ 6

Fraction PFA significantly decreased the mean onset of sleep [F (3, 20) = 12.572, P < 0.001] without affecting the total duration [F (3, 20) = 2.213, P = 0.118] (Figure 3B). Fraction PFB non-dose-dependently increased the total sleep duration (Figure 3C) while fraction PFC dose-dependently increased the total sleep duration (Figure 3D). Bergenin, similar to the PFA from where it was isolated, significantly decreased the mean onset of diazepam induced sleep [F (2, 15) =7.167; P < 0.01] without affecting the total sleep duration [F (2, 15) = 0.090, P=0.914] (Figure 4). 

**Figure 5:**
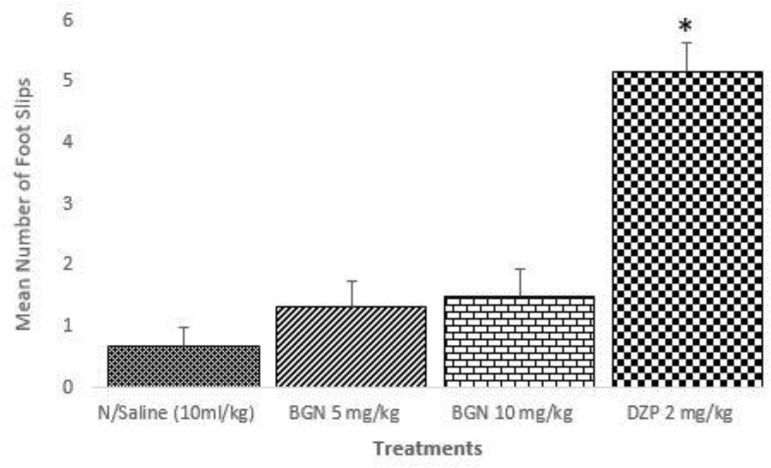
Effect of Bergenin (BGN) and Diazepam (DZP) on motor coordination in mice *p < 0.001 compared to control; n ═ 6

In the beam walking assay, while bergenin did not affect the number of foot slips, diazepam (2 mg/kg) significantly increased the total number of foot slips (Figure 5).

## Discussion

The present study reports the isolation of bergenin from the root of *S. virosa* and its sleep inducing property, for the first time. Bergenin has been isolated from several plants (See Patel et al., 2012 ▶ for a review). It has also been isolated from the leaves of *S. virosa* (Sanogo et al., 2009 ▶). Its isolation from the root of the plant in this study suggests that it is also an important phytochemical found in *S. virosa* in addition to the securinine alkaloids. Bergenin isolated from different plants have been found to possess anti-oxidant (Takahashi et. al., 2003 ▶), anti-inflammatory (Nazir et al., 2007 ▶), anti-retroviral (Piacente et al., 1996 ▶), anti-arrythmic (Pu et al., 2002 ▶) and hepato-protective (Lim et al., 2000 ▶) properties. However, to the best of our knowledge, there is no data in the literature on the effect of bergenin on the central nervous system.

Diazepam-induced sleep model has been used as a preliminary method to study CNS active compounds (Danjuma et al., 2013 ▶). Compounds that decrease the onset of sleep have sleep-inducing property while those that increase the total diazepam sleep duration are known to produce general CNS depression (Perez, 1998 ▶; Rakotonirina et al., 2001 ▶). Previously, the crude methanolic extract of root bark of *S. virosa* has been reported to affect both indices of sleep (onset and duration) in diazepam-induced sleep paradigm (Magaji et al., 2008 ▶). Conversely, the butanol fraction of the crude extract was found to decrease the sleep onset without significantly affecting the total sleep duration (Magaji et al., 2012 ▶). In this study, we found out that bergenin behaved similar to the n-butanol fraction from which it was isolated by decreasing the onset of sleep without significantly affecting the total sleep duration, suggesting that it may contribute to the sleep-inducing property of the extract, since other phytochemical such as flavonoids, saponins and alkaloids found in the extract and fraction have been reported to have sleep-modulating properties. The roles of various endogenous neurotransmitters in the sleep have been well documented. The dopaminergic neurotransmission is involved in the maintenance of behavioral alertness and waking mechanism while a decrease in dopaminergic receptor activity has been found to produce a state of sleepiness (Osuide and Wambebe, 1980 ▶; Gillin et al., 2000 ▶). Dopamine D_2_ receptor have been reported to play an essential role in the maintenance of wakefulness but not in homeostatic regulation of non-rapid eye movement sleep (NREM) (Qu et al., 2010 ▶). Conversely, the dopamine D_1_ receptors are involved in the regulation of rapid eye movement (REM) sleep process (Trampus et al., 1991 ▶). The benzodiazepines produce their sedative, anxiolytic, muscle relaxant, anticonvulsant as well as cognitive impairment through potentiation of GABAergic neurotransmission. Specifically, the benzodiazepines are known to allosterically modulate the a_1 _subtype of the GABA_A_ receptor to produce their sedative effect (McKernan et al., 2000 ▶). 

The exact mechanism via which bergenin reduces the mean onset of diazepam-induced sleep was not elucidated here. However, the activity of bergenin in reduction of sleep onset may be similar to that of ramelteon, a chronohypnotic that acts on the melatonin MT_1_ and MT_2_ receptors in the suprachiasmatic nucleus which promotes sleep onset without the characteristic side effects (cognitive impairment, motor disturbance, dependence, tolerance, hangover, and rebound insomnia) associated with the GABA_A_ receptor modulating agents (Miyamoto, 2009 ▶). 

 Warfarin, a coumarin analogue was found to increase hexobarbital-induced sleeping time (Apseloff et al., 1991 ▶). Conversely, the 4-hydroxycoumarin isolated from the* Viola betonicifolia *was found to be devoid of sleep potentiating property (Muhammad et al., 2013 ▶), suggesting that the CNS activity of coumarins may not be general but seems to be dependent on the chemistry of the compounds. Coumarin and derivatives have been reported to be neuroprotective (Kang et al., 2005 ▶; Kang and Kim, 2007 ▶; Epifano et al., 2008 ▶). Bergenin has also been reported to possess antioxidant and anti-inflammatory potentials (Takahashi et. al., 2003 ▶; Nazir et al., 2007 ▶) and could therefore possess neuroprotective activity. 

The Beam walking assay is a more sensitive tool than the rota rod in determination of benzodiazepine-induced motor coordination deficit (Stanley, 2005 ▶). Compounds that induce motor coordination deficit are known to increase the number of foot slips when compared to the control. The time to complete the task and the number of falls are not as sensitive as the number of foot slips. The inability of bergenin to increase the number of foot slips suggest that it may not interfere with cerebellar-dependent motor coordination at the doses used in the study (Otte et al., 2009 ▶); and that its action may be limited to the limbic area of the brain that control arousal. 

In conclusion, bergenin isolated from the root bark of *S. virosa *possesses sleep-inducing property. Study is currently going on to predict the possible mechanism (s) by which bergenin influences the CNS using different behavioral paradigms of neuropsychiatric disorders, some of which have oxidative imbalance and neuronal excitation pathologies. 
